# Prevalence of tick-borne pathogens in questing *Ixodes ricinus* ticks in urban and suburban areas of Switzerland

**DOI:** 10.1186/s13071-017-2500-2

**Published:** 2017-11-09

**Authors:** Corinne P. Oechslin, Daniel Heutschi, Nicole Lenz, Werner Tischhauser, Olivier Péter, Olivier Rais, Christian M. Beuret, Stephen L. Leib, Sergei Bankoul, Rahel Ackermann-Gäumann

**Affiliations:** 1 Spiez Laboratory, Federal Office for Civil Protection, Austrasse, Spiez, Switzerland; 2 Institute for Infectious Diseases, University of Bern, Friedbühlstrasse, Bern, Switzerland; 3 Graduate School for Cellular and Biomedical Sciences, University of Bern, Bern, Switzerland; 4 ZHAW Life Science and Facility Management, Grüental, Wädenswil, Switzerland; 5 retired, Infectious Diseases, Central Institute of Valais Hospitals, Sion, Switzerland; 6 Laboratory of Ecology and Evolution of Parasites, Institute of Biology, University of Neuchâtel, Emile Argand, Neuchâtel, Switzerland; 7 Medical Services Directorate, Swiss Armed Forces, Ittigen, Switzerland; 8 Swiss National Reference Centre for tick-transmitted diseases, Spiez, Switzerland

**Keywords:** *Ixodes ricinus*, *Borrelia*, *Rickettsia*, *Anaplasma*, "*Candidatus* Neoehrlichia mikurensis", *Babesia*, Tick-borne encephalitis virus, "*Candidatus* Midichloria mitochondrii", Urban, NGS

## Abstract

**Background:**

Throughout Europe, *Ixodes ricinus* transmits numerous pathogens. Its widespread distribution is not limited to rural but also includes urbanized areas. To date, comprehensive data on pathogen carrier rates of *I. ricinus* ticks in urban areas of Switzerland is lacking.

**Results:**

*Ixodes ricinus* ticks sampled at 18 (sub-) urban collection sites throughout Switzerland showed carrier rates of 0% for tick-borne encephalitis virus, 18.0% for *Borrelia burgdorferi* (*sensu lato*), 2.5% for *Borrelia miyamotoi*, 13.5% for *Rickettsia* spp., 1.4% for *Anaplasma phagocytophilum*, 6.2% for "*Candidatus* Neoehrlichia mikurensis", and 0.8% for *Babesia venatorum* (*Babesia* sp., EU1). Site-specific prevalence at collection sites with *n* > 45 ticks (*n* = 9) significantly differed for *B. burgdorferi* (*s.l.*), *Rickettsia* spp., and "*Ca.* N. mikurensis", but were not related to the habitat type. Three hundred fifty eight out of 1078 *I. ricinus* ticks (33.2%) tested positive for at least one pathogen. Thereof, about 20% (71/358) were carrying two or three different potentially disease-causing agents. Using next generation sequencing, we could detect true pathogens, tick symbionts and organisms of environmental or human origin in ten selected samples.

**Conclusions:**

Our data document the presence of pathogens in the (sub-) urban *I. ricinus* tick population in Switzerland, with carrier rates as high as those in rural regions. Carriage of multiple pathogens was repeatedly observed, demonstrating the risk of acquiring multiple infections as a consequence of a tick bite.

**Electronic supplementary material:**

The online version of this article (10.1186/s13071-017-2500-2) contains supplementary material, which is available to authorized users.

## Background

*Ixodes ricinus* is the most frequent tick species throughout Europe. Its life-cycle proceeds through three developmental stages, larvae hatching from eggs, nymphs, and adult males or females. *Ixodes ricinus* may act as a parasite on more than 200 different species, including humans. It serves as a vector for numerous human and animal pathogens of bacterial, viral, or protozoic origin [1, 2].

Tick-borne encephalitis virus (TBEV) causes disease of variable severity, ranging from subclinical infections to severe disease with neurological involvement and potentially fatal outcome. TBEV is taxonomically classified into European, Siberian and Far Eastern subtypes; *I. ricinus* is the principal vector for the European subtype of the virus [3, 4]. Multiple species of rodents, insectivores and carnivores serve as reservoir hosts of TBEV [5, 6]. Although the virus is transmitted transovarially in *I. ricinus* ticks, this transmission is not effective enough in sustaining viral circulation in nature [7]. Co-feeding is essential for TBEV maintenance in natural foci [8]. Mean prevalence in endemic regions ranges from < 0.1 to 5% in Europe and 4 to 39% in Asia [9]. In Switzerland, 38/165 rural sites screened for the presence of TBEV in *I. ricinus* ticks were shown to harbor natural foci, with a mean virus prevalence of 0.46% [10].

Lyme borreliosis is a multisystemic disease that causes local infections in the skin or disseminates to various tissues, including joints, the central nervous system and the heart [11]. It is prevalent in North America, Europe, parts of North Africa, and northern Asia. Within the *B. burgdorferi* (*sensu lato*) complex, *B. afzelii*, *B. burgdorferi* and *B. garinii* are confirmed agents of localized, disseminated and chronic manifestations of Lyme borreliosis, whereas *B. spielmanii*, *B. bissettii* and *B. valaisiana* have only been associated with few cases of Lyme borreliosis [11]. *Ixodes ricinus* is the predominant vector of *B. burgdorferi* (*s.l.*) in Europe and small mammals and ground-foraging birds serve as reservoir hosts [2, 12]. Transovarial transmission of *B. burgdorferi* (*s.l.*) in *I. ricinus* is limited [13]. Mean carrier rates are higher in adults (18.6%) than in nymphs (10.1%), and highest carrier rates are found in central Europe [14]. In questing *I. ricinus* ticks in (sub-) urban areas of Europe, carrier rates range between 2 and 40.8% [2]. In rural areas of Switzerland, prevalence ranges between 9 and 40% for nymphs and from 22 to 47% in adults [15].

*Borrelia miyamotoi* may cause a febrile illness possibly presenting as relapsing fever. In immunocompromised patients, it may cause severe disease including meningoencephalitis. The prevalence of *B. miyamotoi* in *I. ricinus* ticks in Europe ranges between 0 and 4%. In urban areas of France, a prevalence of 4% was found, whereas the carrier rate was much lower (2/428) in a study conducted in peri-urban and urban areas in southern England [16–20]. Potential reservoir hosts include species of rodents and birds. Different tick species such as *Ixodes scapularis* and *I. ricinus* transmit *B. miyamotoi* transovarially [13, 18, 21–23].

Various *Rickettsia* species are transmitted by hard ticks in Europe, including *R. helvetica*, *R. monacensis*, *R. conori* and *R. slovaca* implicated in human disease [2, 24, 25]. In Switzerland, *R. helvetica* and *R. monacensis* appear to be of particular importance [26, 27]. Clinical signs of infections with *R. helvetica* include fever, headache and myalgia [28]. *Rickettsia monacensis* may cause Mediterranean spotted-fever like illness [29]. The prevalence of *R. helvetica* and *R. monacensis* in *I. ricinus* ticks in Europe ranges from 0.5 to 66%, or 0.5 to 34.5%, respectively [2, 30–32]. In Germany and Slovakia, prevalence of *Rickettsia* spp. in urban sites ranged between 2.2 and 30.1% [30, 31, 33, 34]. Ticks serve as both the vector and main reservoir of *Rickettsia* spp., with transstadial and transovarial transmission being documented [2].

*Anaplasma phagocytophilum* causes disease in domestic ruminants and horses [35], but may also infect other mammalian species, including humans [36]. Clinical manifestation in humans ranges from mild self-limiting febrile illness to fatal infections [36–39]*. Anaplasma phagocytophilum* is not transmitted transovarially in *I. ricinus* ticks [40]. Its epidemiological cycles involving mammalian hosts and vectors are complex and comprise different bacterial ecotypes. Carrier rates of *I. ricinus* in Europe range between < 1% and about 20% [36]. At urban sites (Austria, France, Slovakia, Hungary), carrier rates between 0.7 and 8.8% have been documented [20, 41–44].

"*Candidatus* Neoehrlichia mikurensis" has been detected in *I. ricinus* ticks in various European countries, with carrier rates ranging from 0.95 to 23.5% [42, 45–48]. The reservoir role of several rodent species has been proven [49–52]; transovarial transmission in *I. ricinus* has not yet been reported [2]. In urban habitats in Slovakia, "*Ca.* N. mikurensis" has been detected in both *I. ricinus* ticks and rodents, with prevalence in *I. ricinus* ranging between 1.0–2.4% [44, 53]. Only a limited number of severe human disease cases associated with fever, septicemia, malaise and weight loss have been described so far, most often but not exclusively affecting patients with immune deficiency [54–57].

*Babesia* spp. are best known to cause animal illness. Three species are currently recognized to be involved in human disease in Europe: *B. divergens*, *B. venatorum* (*Babesia* sp., EU1), and *B. microti*, with the bovine parasite *B. divergens* being thought to be responsible for most cases. Clinical signs of babesiosis such as flu-like symptoms or hemolytic anemia are usually but not exclusively limited to immunocompromised patients [2, 58, 59]. Carrier rates of *I. ricinus* ticks in Europe range around 0.2 to 3.0% for *B. divergens* and 0.4 to 1.3% for *B. venatorum* [2, 60–62]. There is evidence for circulation of *B. divergens* and *B. venatorum* in urban areas, given that the respective host species (cattle, ungulates) are present [2, 30, 63]. In Germany, Poland and Slovakia, prevalence in urban habitats ranges from 0.4 to 4.5% [33, 64, 65]. In rural areas of Switzerland, a prevalence of 1.9% has been documented [27]. *Babesia* spp. are generally known to be transmitted both transstadially and transovarially in ticks [66]. However, transovarial transmission could so far not be experimentally demonstrated for *B. microti* [67].

In Switzerland, several studies on the prevalence of all of the above-described tick-borne pathogens in questing ticks have been performed [10, 27, 68–73]. However, data on the carrier rate of ticks in suburban areas of Switzerland are scarce [60, 74], and data on tick-borne pathogens in questing ticks in urban areas were not available to date. In this study, we analyzed 1078 questing *I. ricinus* ticks sampled at (sub-) urban collection sites throughout Switzerland for the presence of TBEV, *B. burgdorferi* (*s.l.*), *B. miyamotoi*, *Rickettsia* spp., *A. phagocytophilum*, "*Ca.* N. mikurensis" and *Babesia* spp. Additionally, we analyzed ten tick DNA samples using next generation sequencing (NGS), including two positive samples as well as eight randomly selected samples negative for the investigated pathogens. In these latter eight samples, we searched for pathogens potentially missed using specific screening PCRs as well as for members of the tick microbiota.

## Methods

### Tick sampling

A total of 45 (sub-) urban study areas were defined in collaboration with the respective authorities. Within the areas, similar collection sites of at least 100 square meters were chosen. Collection sites in urban parks, river sides, cemeteries or open air swimming pool areas were characterized by the presence of bushes or trees and some kind of litter layer. Within urban forests surrounded by built-up areas and within suburban forests located at the border of the city, collection sites were situated at the edge of deciduous forest with high recreational frequentation. Sampling was performed between 10:00 am and 16:00 pm but not on rainy days. Most collection sites were visited only once in June 2016, with a monthly average temperature of about 16 °C. At 11 collection sites in the city of Zürich, ticks were collected throughout the year at 6 different time points (June, July, September and November 2015, April and May 2016). These sites were selected to be visited several times in the framework of another study, where the presence of ticks was related to the number of registered tick bites (unpublished data). Temperature at the collection days for these sites ranged between 13–30 °C, with a relative humidity ranging between 50–85%. Ticks were collected by flagging low vegetation using a terry towel of 1 m of width and length fixed to a wooden stick. Time invested for tick collection at one collection site ranged between 3 and 5 h. Tick collection was not standardized, since this study did not focus on the tick density in the investigated sites, but rather on the pathogen prevalence found within the analyzed ticks. Collected ticks were kept alive at 4 °C. Following identification based on morphological characteristics [75, 76], ticks were individually sorted into collection microtubes (Qiagen, Hilden, Germany) and stored at -20 °C.

### Sample preparation

Tick samples were homogenized in 600 μl of pre-cooled PBS using the TissueLyser system (Qiagen, Hilden, Germany). After a short centrifugation step, 400 μl of the supernatant were transferred to a Deepwell plate (Eppendorf, Hamburg, Germany), 60 μl of glycerin were added per well and the plates stored at -80 °C for further use. 100 μl of the supernatant were used for nucleic acid extraction.

### Nucleic acid (NA) extraction

100 μl of tick homogenate supernatant were lysed in 400 μl of AVL buffer supplemented with InhibitEX Tablets (Qiagen, Hilden, Germany) in a 96-well MagNA Pure processing cartridge (Roche, Penzberg, Germany). NA extraction was performed with the MagNA Pure 96 instrument and the MagNA Pure 96 DNA and Viral NA Large Volume kit, using the Pathogen Universal LV 2.0 protocol, a sample volume of 500 μl and an elution volume of 100 μl. NA quality was randomly controlled using the Agilent 2100 Bioanalyzer system with the Agilent High Sensitivity DNA Kit (Agilent Technologies Inc., Santa Clara, California, USA).

### Real-time (reverse transcription-) PCR

The real-time (RT-PCR) systems used for screening the tick samples on the presence of TBEV, *Borrelia* spp., *B. miyamotoi*, *Rickettsia* spp., *A. phagocytophilum*, "*Ca.* N. mikurensis" and *Babesia* spp. are summarized in Table 1. For *Rickettsia* spp. and *Babesia* spp., two screening systems were used.

**Table 1:** Real-time (RT-PCR) systems used for screening tick samples for the presence of various pathogens

Pathogen	Primer sequences (5'–3')	Amplicon length (bp)	PCR cycler^a^	Mastermix	Protocol	Reference primer sequences
*A. phagocytophilum*	locus: major surface protein 2 (msp2) gene (forward: ATG GAA GGT AGT GTT GGT TAT GGT ATT; reverse: TTG GTC TTG AAG CGC TCG TA; probe: FAM-TGG TGC CAG GGT TGA GCT TGA GAT TG-BHQ1)	75	AB QuantStudio 12K Flex	LightCycler Multiplex DNA Master & 50 nM external ROX (final primer conc.: 0.5 μM; final probe conc.: 0.25 μM; reaction volume: 10 μl; sample volume: 2.5 μl)	Initial denaturation/polymerase activation (95 °C, 30 s); 2-step-amplification (45× 95 °C, 5 s; 60 °C, 30 s); cooling (40 °C, 30 s)	[108]
*Babesia* spp.	locus: 18S ribosomal RNA gene (forward: TGA ACG AGG AAT GCC TAG TATG; reverse: CCG AAT AAT TCA CCG GAT CAC TC; probe: FAM-AAG TCA TCA GCT TGT GCA GAT TAC GTC CCT-BHQ1)	116	AB QuantStudio 12K Flex	LightCycler Multiplex DNA Master & 50 nM external ROX (final primer conc.: 0.5 μM; final probe conc.: 0.25 μM; reaction volume: 10 μl; sample volume: 2.5 μl)	Initial denaturation/polymerase activation (95 °C, 30 s); 2-step-amplification (45× 95 °C, 5 s; 60 °C, 30 s); cooling (40 °C, 30 s)	[65]
*B. microti*	locus: 18S ribosomal RNA gene (forward: CAG GGA GGT AGT GAC AAG AAA TAA CA; reverse: GGT TTA GAT TCC CAT CAT TCC AAT; probe: FAM-TAC AGG GCT TAA AGT CT-MGBNFQ)	71	AB QuantStudio 12K Flex	LightCycler Multiplex DNA Master & 50 nM external ROX (final primer conc.: 0.5 μM; final probe conc.: 0.25 μM; reaction volume: 10 μl; sample volume: 2.5 μl)	Initial denaturation (95 °C, 30 s); 2-step-amplification (45× 95 °C, 5 s; 60 °C, 30 s); cooling (40 °C, 30 s)	[109]
*Borrelia* spp.	locus: 16S ribosomal RNA gene (forward: GGTCAAGACTGACGCTGAGTCA; reverse: GCGGCACACTTAACACGTTAG; probe: FAM-TCT ACG CTG TAA ACG ATG CAC ACT TGG TG-BHQ1)	136	Roche LightCycler 96	TaqMan Fast Advanced Master Mix (final primer conc.: 0.4 μM; final probe conc.: 0.25 μM; reaction volume: 25 μl; sample volume: 5 μl)	Uracil-N glycosylase incubation (50 °C, 120 s); initial denaturation/polymerase activation (95 °C, 20 s); 2-step-amplification (45× 95 °C, 3 s; 60 °C, 30 s)	[110]
*B. miyamotoi*	locus: 16S ribosomal RNA gene (forward: CGC TGT AAA CGA TGC ACA CTT GGT GTT AAT C; reverse: CGG CAG TCT CGT CTG AGT CCC CAT CT;probe: FAM-CCT GGG GAG TAT GTT CGC AAG AAT GAA ACT C-BHQ1)	352	Roche LightCycler 96	TaqMan Fast Advanced Master Mix (final primer conc.: 0.4 μM; final probe conc.: 0.25 μM; reaction volume: 25 μl; sample volume: 5 μl)	Uracil-N glycosylase incubation (50 °C, 120 s); initial denaturation/polymerase activation (95 °C, 20 s); 2-step-amplification (45× 95 °C, 3 s; 60 °C, 30 s)	[19]
"*Ca.* Neoehrlichia mikurensis"	locus: 16S ribosomal RNA gene (forward: ATC CTG GCT CAG AAC GAA CG; reverse: TGA TCG TCC TCT CAG ACC AGC; probe: FAM-ACC CAT AGT AAA CTA CAG CTA CA-MGBNFQ)	280	AB QuantStudio 12K Flex	LightCycler Multiplex DNA Master & 50 nM external ROX (final primer conc.: 0.5 μM; final probe conc.: 0.25 μM; reaction volume: 10 μl; sample volume: 2.5 μl)	Initial denaturation/polymerase activation (95 °C, 30 s); 2-step-amplification (45× 95 °C, 5 s; 60 °C, 30 s); cooling (40 °C, 30 s)	[99]
*Rickettsia* spp.	locus: citrate synthase-encoding gene (gltA) (forward: TCG CAA ATG TTC ACG GTA CTT T; reverse: TCG TGC ATT TCT TTC CAT TGT G; probe: FAM-TGC AAT AGC AAG AAC CGT AGG CTG GAT G-BHQ1)	74	AB QuantStudio 12K Flex	LightCycler Multiplex DNA Master & 50 nM external ROX (final primer conc.: 0.5 μM; final probe conc.: 0.25 μM; reaction volume: 10 μl; sample volume: 2.5 μl)	Initial denaturation/polymerase activation (95 °C, 30 s); 2-step-amplification (45× 95 °C, 5 s; 60 °C, 30 s); cooling (40 °C, 30 s)	[26]
*R. helvetica*	locus: 23S ribosomal RNA gene (forward: TTT GAA GGA GAC ACG GAA CAC A; reverse: TCC GGT ACT CAA ATC CTC ACG TA; probe: FAM-AAC CGT AGC GTA CAC TTA-MGBNFQ)	65	AB QuantStudio 12K Flex	LightCycler Multiplex DNA Master & 50 nM external ROX (final primer conc.: 0.5 μM; final probe conc.: 0.25 μM; reaction volume: 10 μl; sample volume: 2.5 μl)	Initial denaturation/polymerase activation (95 °C, 30 s); 2-step-amplification (45× 95 °C, 5 s; 60 °C, 30 s); cooling (40 °C, 30 s)	[26]
TBEV	locus: envelope gene (forward: GGT TTG TGA GGC AAA AAA GAA; reverse: TCC CGT GTG TGG TTC GAC TT; probe: FAM-AAG CCA CAG GAC ATG TGT ACG ACG CC-BHQ1)	88	AB QuantStudio 12K Flex	LightCycler Multiplex RNA Virus Master & 50 nM external ROX (final primer conc.: 0.5 μM; final probe conc.: 0.25 μM; reaction volume: 10 μl; sample volume: 2.5 μl)	Reverse transcription (50 °C, 600 s); initial denaturation (95 °C, 30 s); 2-step-amplification (45× 95 °C, 5 s; 60 °C, 30 s); cooling (40 °C, 30 s)	[10]

^a^Pipetting of Roche LightCycler 96 well plates was done using the QIAgility system (Qiagen); for pipetting of MicroAmp Optical 384 well plates the Hamilton Microlab Star was used

### Sanger (capillary electrophoresis) sequencing

Samples positive for *Borrelia* spp., *Rickettsia* spp. and *Babesia* spp. were further examined by sequence analyses to identify the respective species. A subset of samples, where tick species identification based on morphological characteristics was unclear (mainly larvae, *n* = 75), were analyzed by Sanger sequencing as well. Nested PCR amplifications and sequence analyses were done by Microsynth (Balgach, Switzerland) using the primers and annealing temperatures summarized in Table 2. First-step PCR reactions were run with 2.5 μl of template DNA in a total volume of 12.5 μl including 0.5 μM of each primer, 200 μM dNTPs, 1.5 mM MgCl_2_ and 0.02 U/μl KAPA2G Robust polymerase (Axon Lab, Baden, Switzerland). Fourty cycles were run for each PCR (denaturation: 20 s, 95 °C; annealing: locus-specific temperatures, 20 s; elongation: 100 s, 72 °C; final elongation step: 45 s, 72 °C). First-step PCR products were diluted 1:100 for the second-step PCRs, which were run under the same PCR conditions as described for the first-step PCR using the nested primers described in Table 2. Successful amplification was verified on a 1.5% agarose gel. PCR products were purified and uni-directionally Sanger sequenced. Sequences were quality-trimmed and manually edited, then locus-wise subjected to alignment and phylogenetic analysis using the Phylogeny.fr website [77]. Species identification was done using BLASTn comparison (NCBI nucleotide database) [78, 79]. The sequences obtained from this study have been deposited in the GenBank database (MF121944–MF121977).

**Table 2:** Primer sequences and annealing temperatures of Sanger sequencing reactions

Species	Primer sequences (5'–3')	Amplicon length (bp)	Amplicon position (reference sequence)	Annealing T (°C)	Reference
Fist-step PCR					
*Borrelia* spp.	locus: 5S-23S intergenic spacer (forward: GAG TTC GCG GGA GAG TAG GTT ATT; reverse: TCA GGG TAC TTA GAT GGT TCA CTT CC	420	3063–3483 (JX564636.1)	64	[111]
*Babesia* spp.	locus: 18S ribosomal RNA gene (forward: GTC TTG TAA TTG GAA TGA TGG; reverse: TAG TTT ATG GTT AGG ACT ACG)	489	466–955 (AJ439713)	58	[112]
*Rickettsia* spp.	locus: 23S–5S intergenic spacer (forward: GAT AGG TCR GRT GTG GAA GCA C; reverse: TCG GGA YGG GAT CGT GTG TTT C)	388	1–388 (AY125012)	68	[113]
Ticks	locus: cytochrome *c* oxidase (forward: ACW AAY CAY AAA GAC ATT GGA AC; reverse: WGG ATG CCC RAA RAA TCA AAA T)	704	1242–1946 (KF197132)	48	[114]
Second-step PCR					
*Borrelia* spp.	locus: 23S-5S intergenic spacer (forward: GGA GAG TAG GTT ATT GCC AG; reverse: GGT TCA CTT CCC CTG GTA TC)	396	3073–3468 (JX56436.1)	64	–
*Babesia* spp.	locus: 18S ribosomal RNA gene (forward: GTA ATT GGA ATG ATG GTG AC; reverse: GTT AGG ACT ACG ACG GAA TC)	475	471–946 (AJ439713)	58	–
*Rickettsia* spp.	locus: 23S-5S intergenic spacer (forward: CAG TAA TGT GTG TAG CTA AC; reverse: ATC GTG TGT TTC ACT CAT GC)	356	22–378 (AY125012)	58	–
Ticks	locus: cytochrome *c* oxidase (forward: AYC AYA AAG ACA TTG GAA CWA T; reverse: GCC CRA ARA ATC AAA ATA RAT G)	686	1245–1933 (KF197132)	48	–

### Reverse line blot (RLB)

Samples positive for *Borrelia* spp. yielding mixed sequences in Sanger sequencing, indicating the presence of multiple *Borrelia* species, were additionally analyzed using RLB as described before [80–83]. The variable spacer region between 2 repeated copies of the 23S and 5S ribosomal genes was amplified by PCR with primers 23S-Bor and B-5S-Bor [80]. For species identification, PCR products were hybridized to 15 oligonucleotide probes [81–83] and blotted on an active Biodyne C membrane using a Miniblotter 45 (Immunetics, Boston, Massachusetts, USA). Hybridization was visualized by incubating the membrane with enhanced chemiluminescence detection liquid and by exposing the membrane to an X-ray film.

### NGS and bioinformatics pipeline

Eight randomly chosen samples negative in all pathogen screening PCRs (samples 3–8), 1 sample positive in *Borrelia* spp., *R. helvetica* and *A. phagocytophilum* screening PCRs (sample 1), and 1 sample positive for *R. helvetica* (sample 2) were subjected to NGS. With these analyses, we aimed to (i) demonstrate the congruency of detecting known pathogens using NGS and real-time screening PCR, (ii) investigate whether some pathogens may potentially be missed using specific screening PCR, and (iii) analyze the microbiota of our *I. ricinus* tick samples. The NGS workflow as well as the bioinformatics pipeline used for data evaluation are described in Additional file 1.

### Pathogen prevalence

Individual carrier rates were assessed for collection sites with more than 45 collected ticks (*n* = 9). Furthermore, since the carrier rates did not significantly differ between habitat types (see statistical analysis), overall prevalence was calculated. Larvae were only included for calculation when the respective pathogen is transmitted transovarially (*B. miyamotoi*, *Rickettsia* spp., *Babesia* spp.). As the samples size for the different dates and collection sites was small, a statistical evaluation of pathogen prevalence in dependence of collection dates was not possible.

### Statistical analysis

The stats package of the R software (version 3.3.2) [84] was used to assess differences in pathogen prevalence between collection sites, habitats (cemetery, urban park, urban forest, suburban forest), developmental stages (larvae, nymphs, adults) and gender (male, female). A generalized linear model (GLM) using the logit link function under the binomial distribution was applied. Chi-square tests were performed to assess significance levels. Pathogen prevalence with respect to developmental stages were analyzed using collection site as an interacting explanatory variable and the Chi-square test was used to compare the main (Stage/Gender*Collection site) model to the reduced model (only collection site). For all analyses, larvae were only included when the respective pathogen is transmitted transovarially (*B. miyamotoi, Rickettsia* spp., *Babesia* spp.). To evaluate the frequency of certain pathogen combinations, a GLM was applied comparing the prevalence of each pathogen in either mono- or multi- (≥ 2) infected ticks. Since only *R. helvetica* were found in larvae, developmental stage was included as an interacting explanatory variable. Finally, Chi-square significance testing was used to compare total count of collected ticks according to season (spring or fall) for sites where multiple collections had been performed. For this purpose a GLM under the Poisson distribution was applied using collection site as an interacting explanatory variable. After applying the Bonferroni correction for multiple comparisons (*n* = 5), a *P-*value < 0.05 was regarded as significant.

## Results

### Tick sampling and species identification

**Fig. 1:**
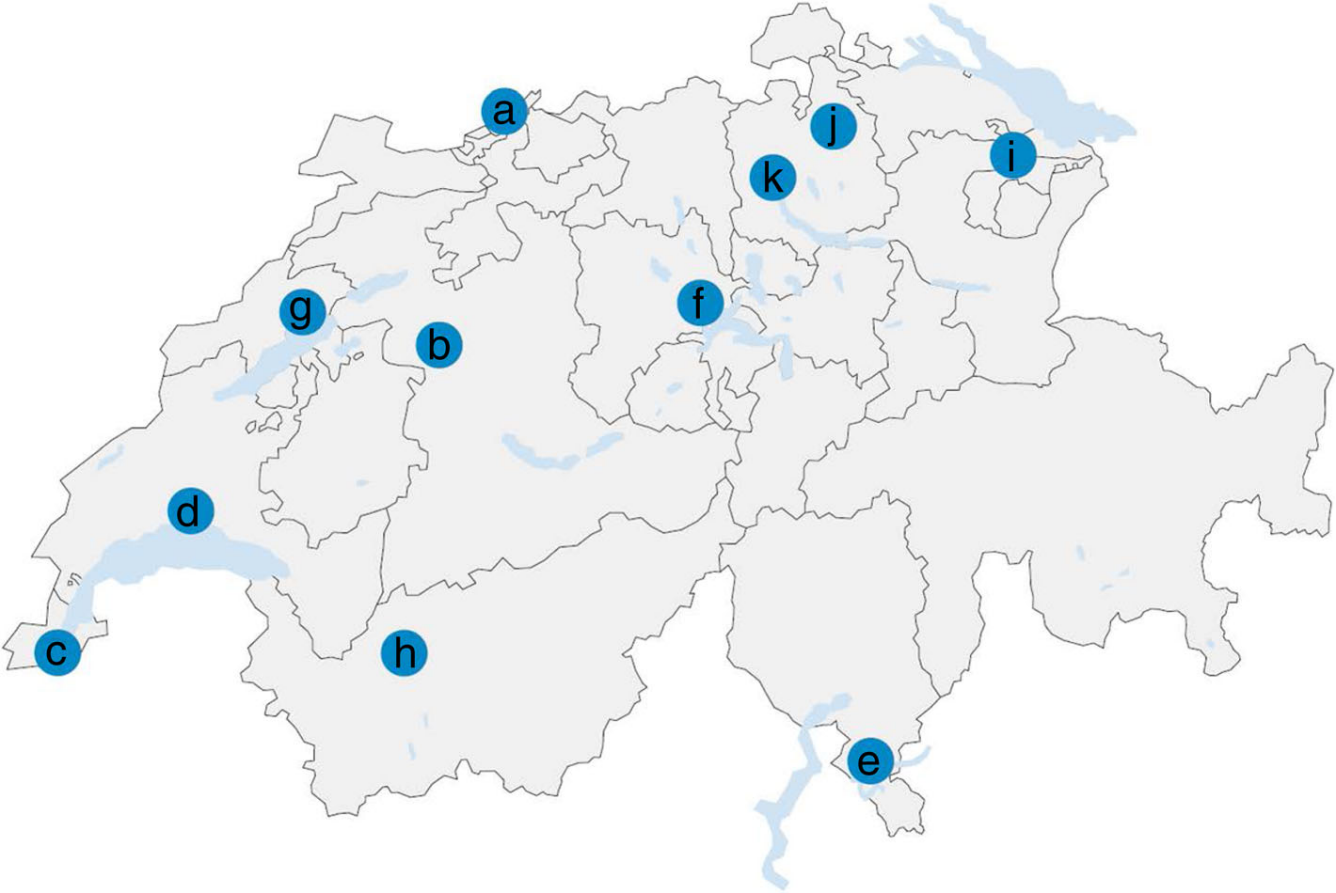
Urban areas in Switzerland analyzed for the presence of pathogens in questing *I. ricinus* ticks. Tick collection was successful at 18 collection sites: (a) Basel (2 sites), (b) Bern (1 site), (c) Geneva (2 sites), (d) Lausanne (1 site), (e) Lugano (1 site), (f) Luzern (1 site), (g) Neuchâtel (2 sites), (h) Sion (1 site), (i) St. Gallen (1 site), (j) Winterthur (1 site), and (k) Zürich (5 sites)

A total of 1,079 ixodid ticks (66 larvae, 740 nymphs, 138 adult males and 135 adult females) were collected at 18 collection sites (Fig. 1, Table 3); at 27 sites, no ticks were found. Tick collection was not standardized with respect to collection time and area, with exception of the sites where flagging was done at multiple time points. Therefore, the collection success in this study must not be equated to questing tick density in the sampling regions. At the collection sites where flagging was done at six different time points, collection was significantly more successful in spring (June 2015, April and May 2016) than in summer or fall (July, September, November 2015) (Chi-square test with Bonferroni correction, *χ* ^2^ = 52.62, *df* = 2, *P* < 0.0001) (Table 4). Except one female *Ixodes hexagonus*, all ticks were identified as *I. ricinus* based on morphological criteria or Sanger sequencing results of the cytochrome *c* oxidase locus.

**Table 3:** Overview of ticks collected at the different collection sites

Collection sites			Number of *I. ricinus* ticks collected^a^					Non-*I. ricinus*
City	Name	Description	Total	Larvae	Nymphs	Adult males	Adult females	
Basel	Friedhof Hörnli	Cemetery	246	1	230	6	9	–
Basel	Margrethenpark	Urban park	83	–	66	10	7	–
Bern	Allmend	Urban park	123	–	9	53	61	–
Bern	Gaswerkareal	Urban park	–	–	–	–	–	–
Bern	Monbijoupark	Urban park	–	–	–	–	–	–
Chur	Schwimmbad obere Au	Open air swimming pool	–	–	–	–	–	–
Chur	Spielplatz Böschengut	Urban park	–	–	–	–	–	–
Geneva	Bois de la Bâtie	Urban forest	–	–	–	–	–	–
Geneva	Bois des frères	Suburban forest	135	1	98	18	18	–
Geneva	Parc des Croppettes	Urban park	2	–	2	–	–	–
Lausanne	Parc de la Gottéttaz	Urban forest	–	–	–	–	–	–
Lausanne	Parc de l’Hermitage	Urban park	103	1	42	32	28	–
Lugano	Gentilino Pambio	Urban forest	–	–	–	–	–	–
Lugano	Parco San Michele	Urban park	1	–	1	–	–	–
Lugano	Parco Del Tassino	Urban park	–	–	–	–	–	–
Lugano	Via degli Abeti	Urban forest	–	–	–	–	–	–
Luzern	Allmend	Urban park	2	–	–	2	–	–
Luzern	Friedhof Friedental	Cemetery	–	–	–	–	–	–
Luzern	Tribschenhorn	Urban park, lake side	–	–	–	–	–	–
Murten	Lindensaal	Urban park	–	–	–	–	–	–
Murten	Stadtgraben	Urban park	–	–	–	–	–	–
Neuchâtel	Jardin du Prince	Urban forest	49	1	44	–	4	1 *I. hexagonus* female
Neuchâtel	Les Cadolles	Suburban forest	115	–	115	–	–	–
Sion	Place de la Planta	Urban park	–	–	–	–	–	–
Sion	Place du Scex	Urban park	–	–	–	–	–	–
Sion	Vissigen	River side	1	–	–	–	1	–
St. Gallen	Bildweiher	Urban park	1	–	–	1	–	–
St. Gallen	Turnhalle Hodlerstrasse	Urban park	–	–	–	–	–	–
Winterthur	Heiligberg	Urban park	3	–	1	1	1	–
Winterthur	Lindengut	Urban park	–	–	–	–	–	–
Winterthur	Rychenbergpark	Urban park	–	–	–	–	–	–
Zürich	Chüeweid, site B-1	Urban park	–	–	–	–	–	–
Zürich	Chüeweid, site B-2	Urban park	–	–	–	–	–	–
Zürich	Chüeweid, site B-3	Urban park	–	–	–	–	–	–
Zürich	Friedhof Sihlfeld, sector C	Cemetery	–	–	–	–	–	–
Zürich	Friedhof Sihlfeld, sector E	Cemetery	2	–	2	–	–	–
Zürich	Limmatufer	River side	–	–	–	–	–	–
Zürich	Rieterpark	Urban park	–	–	–	–	–	–
Zürich	Schärrenwiese, site C-1	Urban park	–	–	–	–	–	–
Zürich	Schärrenwiese, site C-2	Urban park	–	–	–	–	–	–
Zürich	Schärrenwiese, site C-3	Urban park	–	–	–	–	–	–
Zürich	Staudenweg	Suburban forest	5	–	3	1	1	–
*Zürich*	Waldrand Waid, Chäferberg	Suburban forest	47	1	38	6	2	–
*Zürich*	Waidberg Wald	Suburban forest	143	60	74	7	2	–
Zürich	Witikon	Suburban forest	17	1	15	1	–	–

^a^Collection was done in June 2016 at all collection sites except for Waldrand Waid, Chäferberg and Waidberg Wald

**Table 4:** Tick collection success with respect to collection dates for sites with multiple collection attempts

Collection sites		Number of *I. ricinus* ticks collected					
City	Name	June 2015	July 2015	September 2015	November 2015	April 2016	May 2016
Zürich	Waldrand Waid, Chäferberg	8	0	6	0	13	20
Zürich	Waidberg Wald	9	21	19	3	42	49

### Pathogen prevalence

Table 5 summarizes the number of positive *I. ricinus* ticks found per collection site. Pathogen prevalence was not significantly different between collection sites belonging to different habitat types (i.e. cemetery, urban park, urban forest, suburban forest) (*P-*values with Chi-square test using Bonferroni correction *>* 0.1 for *B. burgdorferi* (*s.l.*), *B. miyamotoi*, *A. phagocytophilum* and *B. venatorum*, > 0.05 for "*Ca.* N. mikurensis"). We therefore calculated overall prevalence, which was 0% for TBEV, 18.0% for *B. burgdorferi* (*s.l.*) (8.2% for *B. afzelii*, 1.3% for *B. burgdorferi* (*sensu stricto*), 2.8% for *B. garinii*, 0.9% for *B. valaisiana*, 2.3% for multiple *Borrelia* spp., see below), 2.5% for *B. miyamotoi*, 13.5% for *Rickettsia* spp. (13.2% for *R. helvetica*, 0.3% for *R. monacensis*), 1.4% for *A. phagocytophilum*, 6.2% for "*Ca.* N. mikurensis" and 0.8% for *B. venatorum*. In addition to overall prevalence, we calculated individual carrier rates for collection sites where more than 45 *I. ricinus* ticks had been collected (Table 6). Site-specific carrier rates were was significantly different for *B. burgdorferi* (*s.l.*) (*χ* ^2^ = 50.04, *df* = 8, *P* < 0.0001), *Rickettsia* spp. (*χ* ^2^ = 56.85, *df* = 8, *P* < 0.0001) and "*Ca.* N. mikurensis" (*χ* ^2^ = 27.86 *df* = 8, *P* = 0.006). Pathogen carrier rates did not significantly differ in relevance to tick developmental stages (*P*-values Chi-square test with Bonferroni correction > 0.08). Larvae were exclusively found to be positive for *Rickettsia* spp. at a percentage of 32.8%.

**Table 5:** Pathogen screening results of *I. ricinus* ticks from 18 urban collection sites

Collection sites				Number of *I. ricinus* ticks positive for:											
City	Name	Description	*n*	TBEV	*B.a.*	*B.b.*(*s.s.*)	*B.g.*	*B.va.*	*B.b.*(*s.l.*)	*B.m.*	*R.h.*	*R.m.*	*A.p.*	*B.ve.*	*N.m.*
Basel	Friedhof Hörnli	Cemetery	246	0	15	3	5	1	1	8	37	0	3	5	24
Basel	Margrethenpark	Urban park	83	0	4	2	0	0	0	1	20	0	0	1	0
Bern	Allmend	Urban park	123	0	27	0	7	3	7	2	13	0	4	0	12
Geneva	Bois des frères	Suburban forest	135	0	5	2	10	2	6	4	7	1	3	0	4
Geneva	Parc des Croppettes	Urban park	2	0	0	0	0	0	0	0	1	0	0	0	0
Lausanne	Parc de l’Hermitage	Urban park	103	0	4	1	2	2	1	2	6	2	1	0	4
Lugano	Parco San Michele	Urban park	1	0	0	0	0	0	0	0	0	0	0	0	0
Luzern	Allmend	Urban park	2	0	0	0	0	0	0	0	0	0	0	0	0
Neuchâtel	Jardin du Prince	Urban forest	49	0	2	0	1	1	1	4	2	0	0	0	5
Neuchâtel	Les Cadolles	Suburban forest	115	0	8	0	2	0	6	4	3	0	1	0	4
Sion	Vissigen	River side	1	0	0	0	0	0	0	0	0	0	0	0	0
St. Gallen	Bildweiher	Urban park	1	0	0	0	0	0	0	0	0	0	0	0	0
Winterthur	Heiligberg	Urban park	3	0	1	0	0	0	0	0	2	0	0	0	0
Zürich	Friedhof Sihlfeld, sector E	Cemetery	2	0	0	0	0	0	0	1	0	0	0	0	0
Zürich	Staudenweg	Suburban forest	5	0	2	0	1	0	0	0	2	0	0	0	0
Zürich	Waldrand Waid, Chäferberg	Suburban forest	47	0	7	0	0	0	0	0	11	0	0	0	5
Zürich	Waidberg Wald	Suburban forest	143	0	7	3	0	0	1	1	35	0	1	0	5
Zürich	Witikon	Suburban forest	17	0	1	2	0	0	0	0	3	0	1	0	0

*Abbreviations*: TBEV, Tick-borne encephalitis virus; *B.a., Borrelia afzelii; B.b.*(*s.s.*), *Borrelia burgdorferi* (*s.s.*)*; B.g., Borrelia garinii; B.va., Borrelia valaisiana; B.b.*(*s.l.*)*, Borrelia burgdorferi *(*s.l.*)*, multiple species; B.m., Borrelia miyamotoi; R.h., Rickettsia helvetica; A.p., Anaplasma phagocytophilum; B.ve., Babesia venatorum* (EU1)*; N.m, *"*Candidatus* N. mikurensis"

**Table 6:** Pathogen prevalence in *I. ricinus* ticks collected at 9 urban or suburban collection sites

Collection sites				Prevalence (%)						
City	Name	Description	*n* ^b^	TBEV	*B.b.*(*s.l.*)^a^	*B.m.*	*R.*spp.^a^	*A.p.*	*B.v.*	N.m.^a^
Basel	Friedhof Hörnli	Cemetery	245 (246)	0	17.1	3.3	15.0	1.2	2.0	9.8
Basel	Margrethenpark	Urban park	83	0	7.2	1.2	24.1	0	1.2	0
Bern	Allmend	Urban park	123	0	35.8	1.6	10.6	3.3	0	9.8
Geneva	Bois des frères	Suburban forest	134 (135)	0	18.5	3.0	5.9	2.2	0	3.0
Lausanne	Parc de l’Hermitage	Urban park	102 (103)	0	9.7	1.9	6.7	1.0	0	3.9
Neuchâtel	Jardin du Prince	Urban forest	48 (49)	0	10.2	8.2	4.1	0	0	10.2
Neuchâtel	Les Cadolles	Suburban forest	115	0	13.9	3.5	2.6	0.9	0	3.5
Zürich	Waldrand Waid, Chäferberg	Suburban forest	46 (47)	0	14.9	0	23.4	0	0	10.6
Zürich	Waidberg Wald	Suburban forest	83 (143)	0	14.5	0.7	24.5	1.2	0	6.0

*Abbreviations*: TBEV, Tick-borne encephalitis virus; *B.a., Borrelia burgdorferi* (*s.l.*)*; B.m., Borrelia miyamotoi; R. spp., Rickettsia spp.; A.p., Anaplasma phagocytophilum; B.ve., Babesia venatorum *(EU1)*; N.m, "* *Candidatus* N. mikurensis"

^a^Pathogens with significantly different carrier rates at the different collection sites

^b^The number in parentheses represents the sample size including larvae. Larvae were only included for the calculation of prevalence for pathogens being transmitted transovarially: *B. miyamotoi*, *Rickettsia* spp., *B. venatorum* (EU1)

### Samples with multiple pathogens

**Fig. 2:**
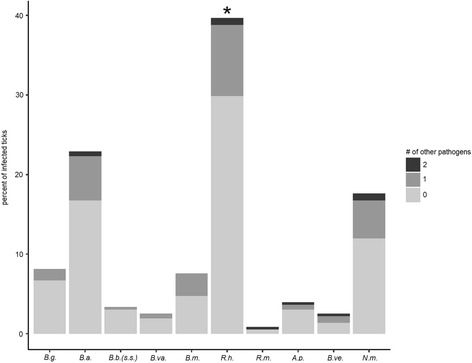
Number of ticks positive for different tick-borne pathogens. The overall height of the bars represents the percentage of infected ticks tested positive for the respective pathogen. The proportions at which the pathogens were detected alone or in combination with one or two others are shown in light gray, dark gray, and black, respectively. *Abbreviations*: *B.g., B. garinii; B.a., B. afzelii; B.b.(s.s.), B. burgdorferi* (*sensu stricto*)*; B.va., B. valaisiana; B.m., B. miyamotoi; R.h., R. helvetica; R.m., R. monacensis; A.p., A. phagocytophilum; B.ve., B. venatorum* (*Babesia* sp., EU1); *N.m.,* "*Candidatus* N. mikurensis"; *B.b.*(*s.l.*), two (or more) different *B. burgdorferi* (*sensu lato*) species. *R. helvetica* was significantly more often detected alone than in association with another pathogen (GLM with developmental stage as a dependent variable; Chi-square test with Bonferroni correction, *P* = 0.023)

**Fig. 3:**
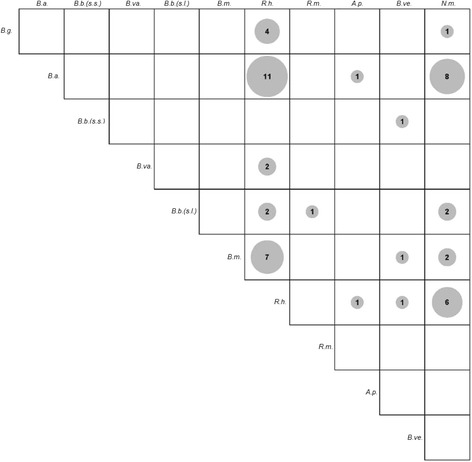
Correlation-plot showing the pathogen combinations observed in urban *I. ricinus* ticks in Switzerland. The more frequent a pathogen combination, the bigger the respective circle in the plot. In addition, the absolute counts of ticks with the particular pathogen combination are given in numbers. *Abbreviations*: *B.g., B. garinii; B.a., B. afzelii; B.b.*(*s.s.*), *B. burgdorferi* (*sensu stricto*)*; B.va., B. valaisiana; B.m., B. miyamotoi; R.h., R. helvetica; R.m., R. monacensis; A.p., A. phagocytophilum; B.ve., B. venatorum* (*Babesia* sp., EU1); *N.m.,* "*Candidatus* N. mikurensis"; *B.b.*(*s.l.*), two (or more) different *B. burgdorferi* (*sensu lato*) species, not distinguishable. Other combinations of three different pathogens are not shown in this plot; these were 1× *B. afzelii* + *R. helvetica* + *A. phagocytophilum* and 1× *B. afzelii* + *B. venatorum* (*Babesia* sp., EU1) + "*Ca.* N. mikurensis"

Out of 1078 ticks, 358 (33.2%) were carrying at least one pathogen. 287 ticks (26.6%) were infected with one, 64 (5.9%) with two, and seven (0.7%) with three different pathogens (Figs. 2, 3). For this analysis, we regarded samples with mixed sequences for *B. burgdorferi* (*s.l.*) in the respective Sanger sequencing reaction as being infected with two different *B. burgdorferi* (*s.l.*) species. The prevalence of *R. helvetica* in mono-infected ticks was significantly higher than the prevalence in multi-infected ticks (Chi-square test with Bonferroni correction, *χ* ^2^ = 9.34, *df* = 2, *P* = 0.023) (Fig. 2).

### RLB for samples with suspected carriage of multiple *B. burgdorferi* (*s.l.*) species

Carriage of multiple *B. burgdorferi* (*s.l.*) species, indicated by mixed sequences in Sanger sequencing analyses, was found in 23 *I. ricinus* ticks (13 nymphs, four males, six females). Using RLB, however, only five of these samples were found to be positive for *B. garinii* and one sample was found to be positive for *B. afzelii*. The remaining 17 samples were negative in RLB analysis. Carriage of multiple *Borrelia* spp. could not be confirmed in any of the samples using RLB.

### NGS

**Fig. 4:**
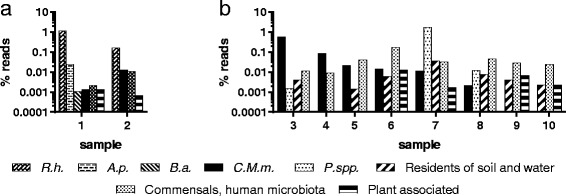
NGS results for 10 *I. ricinus* tick samples. Two samples positive in one or more pathogen screening PCR (**a**) and 8 samples negative in all screening PCRs (**b**) were analyzed. Whole genome amplified samples were sequenced on an Ion S5™, Kraken was used for taxonomic profiling of trimmed reads, and species with low read support were filtered out. Species pathogenic for humans, i.e. *R. helvetica* (*R.h.*)*, A. phagocytophilum* (*A.p.*)*, B. afzelii* (*B.a.*), as well as the tick endosymbiont "*Candidatus* Midichloria mitochondrii" (*C.M.m.*), are represented individually. The remaining species are grouped in *Pseudomonas* spp. (*P.*spp.), other residents of soil and water, commensals, human microbiota, and plant associated bacteria. The bars indicate the percentages of reads assigned to the respective species or groups in a logarithmic scale

NGS was done with a total of ten samples. Although most of the taxonomically classified reads (Kraken output) were assigned to ixodid ticks (96.5–99.9%), which is expected in untreated metagenomics samples of eukaryotes, the read numbers of the pathogens previously identified by screening PCRs were clearly distinguishable from the background noise in sample 1 and 2. The reads assigned to *R. helvetica* denoted 76.3 and 82.6%. The reads of sample 1 classified to *A. phagocytophilum* and *B. afzelii* represented 1.7 and 0.1%, respectively (Fig. 4a). In the remaining eight samples (3–8), no known pathogens could be detected using NGS, which is in agreement with the negative screening PCRs. However, a total of 8 samples (2 adult female, 1 adult male and 5 nymphal ticks) were positive for the tick endosymbiont "*Candidatus* Midichloria mitochondrii" [85] (Fig. 4a, b). In the adult female ticks, the reads classified to "*Ca.* M. mitochondrii" represented 74 and 92% of all bacterial reads. For the male and nymphal ticks, the percentages of bacterial reads classified to this endosymbiont were 0.1% or 0.5–25%, respectively. In addition, every sample contained variable proportions of organisms known to be residents of soil and water, plant associated bacteria, or normal human microbiota (Fig. 4b)

## Discussion

Throughout Europe, *I. ricinus* transmits numerous human and animal pathogens. Its widespread distribution includes urbanized areas. Most wildlife species found in urbanized areas in Europe act as maintenance hosts for *I. ricinus*, but may also serve as reservoirs of tick-borne pathogens. In urban sites, these hosts may be rodents, hedgehogs, shrews, birds, lizards, dogs and cats. In peri-urban areas, larger animals such as foxes, roe deer, and wild boars may act as tick-maintenance and pathogen reservoir hosts. As a consequence of increasing urbanization and the behavior of humans increasingly encroaching on their peri-urban surroundings, the exposition of humans to vector ticks and tick-transmitted pathogens is increasing [2, 86]. Whereas several studies on the prevalence of various tick-borne pathogens in questing *I. ricinus* ticks have been done in Switzerland so far [10, 27, 68–73], only limited research has focused on ticks collected in suburban areas [60, 74]. Here, we analyzed questing *I. ricinus* ticks collected at (sub-) urban sites for the presence of various pathogens. Furthermore, we analyzed ten DNA samples using NGS, thereby detecting true pathogens, tick symbionts, as well as organisms of environmental or human origin.

Tick collection was successful in about 40% (18/45) of the areas flagged during this study. As the focus of our study was the investigation of pathogen prevalence rather than tick density, our tick collection method was not highly standardized. Therefore, the number of collected ticks at the different collection sites does not necessarily reflect the tick density in these areas. As an exception, tick collection effort was approximately standardized at eleven collection sites in the city of Zürich, where tick sampling was done throughout the year at six different time points (June, July, September and November 2015, April and May 2016) (Table 4). When comparing collection success between collections done in spring (April, May, June) to collections done in summer or fall (July, September, November), we found that collection was significantly more successful in spring than in summer or fall (*P* < 0.0001). These findings are in agreement with the results of a study focusing on seasonality of *I. ricinus* ticks on the vegetation in two regions in Switzerland, where a significant decline of questing activity in June was observed [87]. Also, they are explained by the conditions for tick activity (temperature and humidity), which are more likely fulfilled in spring than in summer or fall.

TBEV-infected ticks are distributed in a patchy manner in so-called natural foci. In Europe, within these foci, carriage rates of *I. ricinus* ticks range between < 0.1% and 5% [9] (Switzerland: 0.46% [10]). In the present study focusing on urban areas, we could not detect any TBEV-positive *I. ricinus* ticks. However, given the low expected carrier rates, the sample sizes per collection site are too small to allow for a reliable estimation of TBEV prevalence. Accordingly, the prevalence of 0% has to be interpreted with caution, and more extensive studies are needed to precisely estimate the carrier rate of (sub-) urban *I. ricinus* ticks with TBEV in Switzerland. In studies focussing on urban or peri-urban regions of other European countries (Germany, Poland), TBEV has been detected with carrier rates of 0.31% or 0.1%, respectively [88, 89]. On the other hand, other authors estimate the risk for contracting TBE in urban areas to be low [4].

Four different species belonging to the *B. burgdorferi* (*s.l.*) complex were detected in questing *I. ricinus* ticks in our study: *B. afzelii* (8.2% of ticks), *B. garinii* (2.8%), *B. burgdorferi* (*s.s.*) (1.3%) and *B. valaisiana* (0.9%). All of them are confirmed agents of Lyme borreliosis [11] and have already been detected in *I. ricinus* ticks in other studies in Switzerland [15, 27]. In agreement with previous observations, we found that *B. afzelii* and *B. garinii* are the most prominent species, and that adult ticks are more often infected with *B. burgdorferi* (*s.l.*) than nymphs. The latter observation is explained by the fact that adult ticks had two blood meals with the possibility of acquiring *B. burgdorferi* (*s.l.*), whereas nymphs only had one [14]. The overall prevalence of *B. burgdorferi* (*s.l.*) in the present study was 18.0% (11.7% for nymphs, 25% for adults), with site-specific prevalence being significantly variable (7.2–35.8%, *P* < 0.0001). These observations are in agreement with a study realized in rural areas of Switzerland, where carriage rates ranged between 9–40% for nymphs and from 22 to 47% in adults [15]. The overall prevalence of *B. burgdorferi* (*s.l.*) in questing *I. ricinus* ticks in our study is highly comparable to carriage rates found in urban areas of neighboring countries, ranging from 2.4 to 26.6% in Germany, 10 to 30% in France, and 10.4% in Italy [2, 20, 90–92]. In Sanger sequence analyses of the 5S-23S intergenic spacer region, mixed sequences indicating the presence of multiple *B. burgdorferi* (*s.l.*) species were obtained for 23 samples (13 nymphs, four males, six females). In confirmatory analyses using RLB, however, only 6 of these samples gave positive results (five *B. garinii*, one *B. afzelii*), and carriage of multiple *Borrelia* spp. could not be confirmed in any of the samples. Since many of these samples contained only very small amounts of *Borrelia* DNA with cycle threshold values in screening PCR ranging between 36–40 (data not shown), false-negative results in RLB cannot be excluded. In Sanger sequencing, we were able to raise the sensitivity of the test by adding a second-step PCR using nested primers. Since this was not possible in RLB, we expect this test to have a slightly lower sensitivity, accounting for the discrepancy between RLB and Sanger sequencing results. Therefore, the 23 samples, representing 14.9% of ticks positive for *B. burgdorferi* (*s.l.*), are regarded as being infected with more than one *B. burgdorferi* (*s.l.*) species despite the negative RLB results. This proportion is in agreement with the percentage of carriage of multiple *B. burgdorferi* (*s.l.*) found previously [14].

Human disease cases caused by *B. miyamotoi*, usually presenting as febrile illness have been reported in Russia, USA, the Netherlands and Japan [18]. In *I. ricinus*, the pathogen is found at a prevalence ranging between 0–3.5% in Europe [17–19]. *Borrelia miyamotoi* has been shown to be present in *I. ricinus* ticks in Switzerland in rural areas at a prevalence of about 1% [27]. In our study 2.5% of *I. ricinus* ticks (2.7% of nymphs, 2.6% of adult ticks) were infected with *B. miyamotoi,* which is slightly less than the prevalence described for urban *I. ricinus* ticks in France (4%) [20], but higher than the number of *B. miyamotoi* positive ticks (2/428) reported in a study focusing on urban and peri-urban areas in southern England [16]. Thus, although no disease cases have been reported so far, there is a potential of acquiring such an infection, in urban as well as in rural regions in Switzerland.

Studies investigating *I. ricinus* ticks collected from vegetation or animals in Switzerland revealed *Rickettsia* spp. carriage rates of 7.3 to 14% [21, 26, 93]. In accordance with these results and with the detection of *Rickettsia* spp. in urban areas in other studies in Germany and Slovakia at carrier rates ranging between 2.2–30.1% [30, 31, 33, 34], we found *R. helvetica-*positive *I. ricinus* ticks at a prevalence of 13.2% in urban areas of Switzerland. We observed significant differences in site-specific carrier rates (2.6–24.5%, *P* < 0.0001), which is in agreement with a study in Germany, where prevalence of *Rickettsia* spp. in *I. ricinus* ticks ranged between 0–50% [31]. Unlike the frequent detection of *R. helvetica* in *I. ricinus*, the documentation of human infection with this agent in different countries, including Switzerland, remains rare [25]. In addition to *R. helvetica*, three samples were found to be positive for *R. monacensis*, which has been detected for the first time in Switzerland in 2009 [26] and is known to be present in *I. ricinus* ticks in at least 18 European countries [25]. *R. monacensis* has already been discovered in *I. ricinus* ticks in some urban and peri-urban sites in different European countries [2], which is in accordance with our findings.

*Anaplasma phagocytophilum* has been detected in *I. ricinus* ticks in Europe at a prevalence between < 1% and about 20%. In Switzerland, carrier rates between 1.2–2% have been found [27, 36, 93–97]. Corresponding to these findings we found a carrier rate of 1.4% in urban *I. ricinus* ticks. This rate is in agreement with carrier rates found in urban areas of Austria and France (1.0 and 0.7%, respectively) [20, 41], but is rather low compared to the prevalence found in Slovakia or Hungary (4.5–5.5% and 8.8%, respectively) [42–44]. In Switzerland, human granulocytic anaplasmosis (HGA) is a rarely diagnosed disease so far. However, considering the repeated detection of the causative agents in ticks and knowing that the seroprevalence in humans bitten by *I. ricinus* ticks is 17.1% [98], HGA may increasingly be included in the diagnostic workup of patients with a history of a tick bite. In our study, we merely focused on the detection of *A. phagocytophilum*, without considering the four different ecotypes. So far, all human cases clustered in ecotype I. The different ecotypes are known to have significantly different host ranges, with ecotype I hosts including numerous urban species [2, 50]. We would therefore expect many of the *A. phagocytophilum* isolates detected by real-time PCR in our study to belong to ecotype I. However, the respective analyses have not been done so far.

Neoehrlichiosis is a rare human disease. In Switzerland, a close geographic association of disease cases with *I. ricinus* populations carrying "*Ca.* N. mikurensis" has been shown for the region of Zürich, where pool carrier rates of 0–8% were found [99]. In our study we could confirm the presence of "*Ca.* N. mikurensis" in *I. ricinus* ticks in the region of Zürich, focusing on (sub-) urban areas. In addition, we could show the pathogen to be present in the cities of Basel, Bern, Geneva, and Neuchâtel, with an overall prevalence of 6.2%. This is a higher rate of carriage compared to findings from urban habitats in Slovakia, where prevalence ranged between 1–2.4% [44, 53]. Site-specific carrier rates for "*Ca.* N. mikurensis significantly differed in our study, ranging from 0 to 10.6% (*P* < 0.006). This is in agreement with the variation found in the Swiss study in the rural region of Zürich (pool carrier prevalence between 0–8%) [99]; variable carriage rates ranging between 1.1–4.5% were also found in (sub-) urban habitats in a study conducted in Slovakia, the Czech Republic and Austria [42].

Three *Babesia* species, *B. divergens*, *B. venatorum* and *B. microti* are currently known to cause human disease, and all of them have been found to circulate in urban areas [2, 30, 63]. In 2012 other authors found *Babesia* spp. to be present in 1.9% of *I. ricinus* ticks collected in deciduous forests in Western Switzerland. Thereof, 64.3% were identified as *B. venatorum* and 17.9% as *B. divergens* [27]. Here, we found a carriage rate of questing urban *I. ricinus* ticks of 0.83%. All positive samples were classified as *B. venatorum* using Sanger sequencing. The prevalence of 0.8% is in accordance with *I. ricinus* carrier rates with this parasite in different urban regions in European countries (Germany, Poland and Slovakia), ranging from 0.4% to 4.5% [33, 64, 65]. Since *B. divergens* is a bovine parasite, it would only be expected in areas where cattle are found concurrently with *I. ricinus* ticks [2]. To our knowledge, none of the collection sites of our study represent areas where cattle are present, wherefore the absence of *B. divergens* is plausible. Human babesiosis is a rare but possibly emerging disease in Europe, with about 50 disease cases reported so far [2, 100].

Site-specific pathogen prevalence significantly differed for *B. burgdorferi* (*s.l.*), *Rickettsia* spp., and "*Ca.* N. mikurensis" (*P* < 0.0001 for *B. burgdorferi* (*s.l.*) and *Rickettsia* spp., < 0.006 for "*Ca.* N. mikurensis"). However, these differences were not attributable to the habitat type (i.e. cemetery, urban park, urban forest, suburban forest) (*P >* 0.1 for *B. burgdorferi* (*s.l.*), *B. miyamotoi*, *A. phagocytophilum*, and *B. venatorum*, > 0.05 for "*Ca.* N. mikurensis"). When comparing the carrier rates from our study focusing on (sub-) urban areas to carrier rates found in rural areas of Switzerland, no obvious differences were found for most pathogens (prevalence in urban *vs* rural regions for *B. burgdorferi* (*s.l.*) 18.0 *vs* 9.0–47.0% [15], for *Rickettsia* spp. 13.5 *vs* 7.3–14.0% [21, 26, 93], for *A. phagocytophilum* 1.4 *vs* 1.2–2.0% [27, 36, 93–97], and for "*Ca.* N. mikurensis" 6.2 *vs* 0–8.0% [99]). For *B. miyamotoi*, the overall prevalence was 2.5%, which is higher than the prevalence of about 1% assessed in a study focusing on rural areas of Switzerland. For *Babesia* spp., the overall prevalence assessed in our study focusing on (sub-) urban areas was lower than the prevalence found in the rural area (0.8 *vs* 1.9%) [21]. This latter finding is in agreement with a study comparing the carrier rates between urban and natural habitats in Slovakia [64] and might be in association with the presence of competent reservoir hosts. Altogether, the potential of pathogen transmission as a consequence of a tick bite is highly comparable between urban and rural areas.

In our study, 358 *I. ricinus* ticks (33.2%) were carrying at least one potentially disease-causing agent: 287 (26.6%) were infected with one, 64 (5.9%) with two, and seven (0.7%) with three different pathogens (Figs. 2, 3). In a study investigating about 270 female *I. ricinus* ticks in the French Ardennes, 45% of infected ticks were carrying multiple pathogens [101]. In our study involving *I. ricinus* ticks of all developmental stages, about 80% of infected ticks were positive for only one pathogen, giving a lower proportion of multiple carriage rates. Nevertheless, carriage of multiple pathogens by ticks and therewith co-transmission of pathogens to humans might have important consequences with respect to disease severity and treatment [101–104]. The most frequent pathogen combinations in our study were *B. afzelii* + *R. helvetica* (*n* = 11) and *B. afzelii* + "*Ca.* N. mikurensis" (*n* = 8). Interestingly, the same pathogens have been found to be predominantly involved in coinfections in a study focusing on mixed deciduous forests in the western part of Switzerland. In both, the present and the previous study, *B. afzelii* and *R. helvetica* were the pathogens with the highest prevalence, possibly accounting for the frequent combination of these two bacteria within ticks. *B. afzelii* and "*Ca.* N. mikurensis" share common reservoir hosts, which might account for their concurrent detection in individual *I. ricinus* ticks [27, 105, 106].

Using NGS, we could confirm the presence of all pathogens previously detected by screening PCRs in 2 samples (Fig. 4a, b). In the eight samples negative in all pathogen screening PCRs (samples 3–8), we did not identify any known pathogen using NGS. However, in six of these samples as well as in samples 1 and 2, we could detect the tick endosymbiont "*Ca.* M. mitochondrii", a member of the order *Rickettsiales* (Fig. 4a, b). This bacterium is localized in the mitochondria of ovarian cells in *I. ricinus* female ticks and is transmitted to all offspring. It has been shown to be highly prevalent in *I. ricinus* ticks, with a mean carrier rate of females of 95%, but a lower prevalence in other developmental stages [85, 107]. Our results agree with these findings with both female, but only five out of seven nymphal *I. ricinus* ticks being positive for "*Ca.* M. mitochondrii". Also, the number of reads was much higher in female ticks than in male or nymphal ticks, which is in agreement with the described lower bacterial load in male than in female *I. ricinus* ticks [107].

Besides known pathogens (*R. helvetica*, *A. phagocytophilum*, *B. afzelii*) and tick endosymbionts, we detected various organisms known to be residents of soil and water, plant associated organisms or members of the normal human microbiota in NGS analyses of ten tick samples (Fig. 4a, b). Since we did not wash the surface of the collected ticks prior to sample preparation and nucleic acid extraction, these findings are easily explainable by the presence of these organisms on the exterior of the ticks. While plant, soil and water organisms originate from the collection sites, members of the human microbiota were transmitted to the tick surface during the collection and sorting procedure.

## Conclusions

In this study we documented the presence of *B. burgdorferi* (*s.l.*), *B. miyamotoi*, *R. helvetica*, *R. monacensis*, *A. phagocytophilum*, "*Ca.* N. mikurensis" and *B. venatorum* in the (sub-) urban *I. ricinus* tick population in Switzerland. The pathogen prevalence was as high as the one in rural regions and thus there is a risk of contracting tick-transmitted diseases in urban areas of Switzerland. Carriage of multiple pathogens was observed in about 20% of infected *I. ricinus* ticks, and therefore there is a true risk of acquiring multiple infections as a consequence of a tick bite.

## Additional file

Additional file 1: NGS protocol, bioinformatics pipeline, detailed NGS results and discussion. (DOCX 22 kb)

## Abbreviations

HGA: human granulocytic anaplasmosis
NGS: next generation sequencing
RLB: reverse line blot
TBE: tick-borne encephalitis
TBEV: tick-borne encephalitis virus
