# Supplementary material for: Prevalence of tick-borne pathogens in questing Ixodes ricinus ticks in urban and suburban areas of Switzerland
Source: Parasit Vectors. 2017 Nov 9;10:558. doi: 10.1186/s13071-017-2500-2 (PMC5680829; doi:10.1186/s13071-017-2500-2)
Supplement: Additional file 1: — NGS protocol, bioinformatics pipeline, detailed NGS results and discussion. (DOCX 22 kb) [file 13071_2017_2500_MOESM1_ESM.docx]

**Additional file 1: NGS protocol, bioinformatics pipeline, detailed NGS results and discussion**

**Methods**

**NGS protocol**

During the complete workflow, DNA concentrations were measured using the Qubit® dsDNA High Sensitivity Assay Kit on the Qubit® 2.0 Fluorometer (Thermo Fisher Inc., Waltham, Massachusetts, USA) according to manufacturer’s protocol. Extractives were incubated at room temperature (RT) in the dark overnight to degrade the RNA. The next day, whole genome amplification (WGA) was performed using the PicoPLEX™ WGA Kit (Rubicon Genomics Inc., Ann Arbor, Michigan, USA) according to manufacturer’s protocol for isolated DNA, with a sample volume of 1 μl, corresponding to approximately 50 pg DNA. Final amplification was performed running 16 cycles. Then, WGA products were purified using the QIAquick PCR Purification Kit (Qiagen, Hilden, Germany) with an elution volume of 50 μl. The manufacturer’s protocol was complemented with a) an air drying step at RT after the second wash spin in order to eliminate ethanol rests, b) using Low EDTA TE buffer (1X TE buffer, 10 mM Tris-HCL, pH 8.0, 0.1 mM EDTA) preheated to 39°C as elution buffer and c) extending the elution step to 2 times 1 min. to optimize elution efficiency. The purified WGA products were sheared as described in the Ion Xpress™ Plus gDNA Fragment Library Preparation User Guide, Appendix B. Shearing was performed according to the 400 base-read library size protocol using the Covaris™ M220 Focused‑ultrasonicator (Covaris, Inc., Wobum, Massachusetts, USA) and a DNA sample input of 700 to 1000 ng. The size distribution of the sheared DNA fragments was assessed using the Agilent 2100 Bioanalyzer system with the Agilent High Sensitivity DNA Kit (Agilent Technologies Inc., Santa Clara, California, USA). End-repair, barcoding, and adapter ligation for library preparation was then performed using the AB Library Builder™ System with the Ion Plus Fragment Library Kit (Thermo Fisher Scientific Inc., Waltham, Massachusetts, USA). 400 base-read libraries were prepared according to the Ion Xpress™ Plus and Ion Plus Library Preparation for the AB Library Builder™ System User Guide. As a next step, size-selection of the Ion Torrent™ libraries was performed using the ready-to-use Agencourt® AMPure® XP magnetic bead suspension (Beckman Coulter Eurocenter S.A., Nyon, France). Library samples were mixed with a 0.5X sample volume of bead suspension and incubated at RT for 5 min. The tubes were placed on a magnetic rack (DynaMag™-2 magnet) for 5 min., then the supernatant was transferred to new tubes. Then, the bead suspension was mixed with a 1.8X sample volume, and the tube was processed as described above. The supernatant was discarded and the pellet was washed twice by adding 500 μl of 70 % ethanol to the tube, keeping it on the magnetic rack and rotating it 4 times 180° longitudinally. Ethanol was removed and the bead pellet was air-dried for 5 min. The pellet was resuspended in 20 μl of Low EDTA TE buffer preheated to 39°C and incubated for 2 min. at RT before putting back on the magnetic rack. The supernatant was then transferred to a new tube. Size distribution and quantity of the size-selected libraries were assessed using the Agilent 2100 Bioanalyzer system and the Qubit® 2.0 Fluorometer as described above. Finally, sequencing was done on the Ion S5™ System. Ion 530™ Chips were loaded with 5 equimolar 400 bp libraries at a concentration of 100 pM and Ion S5™ Calibration Standard using the Ion Chef™ System. The run was paused for the Ion Sphere™ Quality Control assay on the Qubit™ 2.0 Fluorometer. Sequencing output ranged between 2,480,455 and 4,049,817 reads per sample.

**Bioinformatics pipeline**

Datasets were checked for quality aspects using FastQC version 0.11.5 [[1](#_ENREF_1)], with an additional search for the WGA adapters. Adapter and quality trimming as well as filtering was performed using Trimmomatic version 0.33, with default parameters modified by a minimum read length of 31 bp [[2](#_ENREF_2), [3](#_ENREF_3)]. The Trimmomatic output ranged between 364,658 and 601,671, which corresponds to an about 4- to 11-fold reduction of the read numbers from pre- to post-trimming due to shortened reads. For taxonomic profiling a custom database of actual reference sequences was built by downloading not anomalous sequences of a) all latest assemblies of ticks, b) viruses, c) all genomes of bacteria in complete status and d) all genomes of bacteria in chromosome status, from the RefSeq [[4](#_ENREF_4)] or Genbank® [[5](#_ENREF_5)] databases. Trimmed and filtered sequencing data were taxonomically classified running Kraken version 0.10.5‑beta [[6](#_ENREF_6)] with default parameters. For the different samples, 62.08 % to 72.49 % of all reads could be assigned to either a tick (61.80 % ‒ 72.27 %) or a viral or bacterial (0.09 % ‒ 2.49 %) reference sequence in the custom database. To reduce false positive hits, species with low read support, in the following called ‘noise’, were filtered out using a histogram showing the abundances of percent reads classified at species level in function of their frequency. The threshold to separate the noise was set at the transition from exponential to plateau phase of the frequency distribution. Species with a percentage of reads above the threshold were considered as true hits. Reads unclassified to the custom database were locally aligned to the NCBI nucleotide database using BLAST version 2.5.0 [[7](#_ENREF_7)]. Further, the trimmed and filtered reads of samples 1 and 2 were mapped to the R. helvetica C9P9 chromosome and plasmid pRhe reference sequences (NZ_CM001467.1, NZ_CM001468.1 respectively) and compared with the Sanger sequencing primers using CLC Genomics Workbench 9.5.2 NGS mapping tools (Qiagen, Hilden, Germany).

**Results**

In addition to known pathogens and endosymbionts (see main manuscript), every sample contained variable proportions of organisms known to be residents of soil and water, plant associated bacteria, or normal human microbiota (Figure 4B). For instance, Lactococcus lactis, a member of the human microbiota, was the most prominent bacterial species in sample 6 (48 % of classified bacterial reads). In sample 7, a high percentage of Pseudomonas spp. (73 %) was detected, with the different species all being residents of soil and water, or partially belonging to the normal human and tick microbiota [[8](#_ENREF_8)]. The reads unclassified by Kraken were further subjected to local alignment in the NCBI nucleotide database, which was successful for 0.6 % – 1.3 % of the reads.

**Discussion – technical aspects of data analysis**

Using NGS we could confirm the presence of all pathogens previously detected by screening PCRs in 2 samples (Figure 4A, B). The WGA step is known to produce bias in the reads output and therewith a falsified proportion of organisms, or even false positive classifications due to polymerase errors. The second effect is partially counteracted by the background reduction of taxonomic profiles, which allows for a narrowing of species to the most probable ones. In our analyses, each species considered as true hit could be related to a biological role in the sample. In contrast, no plausible biological role could be assigned to most of the classified species of the background noise or the alignment hits of unclassified reads (data not shown). The high proportion of unclassified reads yielding no alignment hit most likely represent artificial sequences due to polymerase errors.

**References**

1. FastQC [<http://www.bioinformatics.babraham.ac.uk/projects/fastqc>]. Accessed 15. November 2016

2. Bolger AM, Lohse M, Usadel B: Trimmomatic: a flexible trimmer for Illumina sequence data. *Bioinformatics* 2014, 30(15):2114-2120.

3. Trimmomatic: A flexible read trimming tool for Illumina NGS [<http://www.usadellab.org/cms/?page=trimmomatic>]. Accessed 30. January 2015

4. RefSeq: NCBI Reference Sequence Database [https://[www.ncbi.nlm.nih.gov/refseq/](http://www.ncbi.nlm.nih.gov/refseq/)]

5. GenBank NIH genetic sequence database [https://[www.ncbi.nlm.nih.gov/genbank/](http://www.ncbi.nlm.nih.gov/genbank/)]. Accessed 17. November 2016

6. Wood DE, Salzberg SL: Kraken: ultrafast metagenomic sequence classification using exact alignments. *Genome Biol* 2014, 15(3):2014-2015.

7. NCBI Nucleotide database [https://[www.ncbi.nlm.nih.gov/nucleotide/](http://www.ncbi.nlm.nih.gov/nucleotide/)]

8. Narasimhan S, Fikrig E: Tick microbiome: the force within. *Trends Parasitol* 2015, 31(7):315-323.
